# Validating the family coping questionnaire for eating disorders for caregivers of Japanese patients with eating disorders: association between coping strategies and psychological characteristics

**DOI:** 10.1186/s40337-021-00517-2

**Published:** 2021-12-18

**Authors:** Seraki Miyamoto, Saki Harashima, Kazuhiro Yoshiuchi

**Affiliations:** Department of Stress Sciences and Psychosomatic Medicine, Graduate School of Medicine, The University of Tokyo, Tokyo, Japan

**Keywords:** Coping strategies, Eating disorders, Caregivers’ psychological distress, Family coping strategies, Japan, Anorexia nervosa, Family coping questionnaire

## Abstract

**Background:**

Eating disorders (ED) can adversely affect the psychological health of patients’ caregivers. The present study aimed to validate a Japanese version of the Family Coping Questionnaire for Eating Disorders (FCQ-ED-J) and investigate the association between the coping strategies and psychological states of the caregivers of ED patients.

**Methods:**

The caregivers completed the FCQ-ED-J and the Profile of Mood States. The FCQ-ED measures the coping strategies of caregivers of ED patients to the ED symptom-related behaviors. As confirmatory factor analysis did not yield an adequate model fit, the factor structure of the FCQ-ED-J was analyzed using exploratory factor analysis. Subsequently, the reliability and validity of the FCQ-ED-J were examined using Cronbach’s alpha and Pearson’s correlation coefficients in relation to the Profile of Mood States.

**Results:**

Data from 150 caregivers, including 91 mothers and 34 fathers, was analyzed (mean age 51.1 years, *SD* = 12.0). The FCQ-ED-J, with 13 items grouped across four subscales [“response to binge-eating” (factor 1), “response to frequent weighing” (factor 2), “response to too much physical exercise” (factor 3), and “response to abusing laxatives and/or diuretics” (factor 4)] had Cronbach’s alpha values representing acceptable to good internal consistency (0.71–0.85). Each subscale of the FCQ-ED-J was significantly correlated with the Profile of Mood States subscales.

**Conclusions:**

The FCQ-ED-J had sufficient reliability and validity. The Japanese caregivers’ responses to the patient’s ED symptom-related behavior were associated with their psychological states. Thus, the FCQ-ED-J may offer insight into more effective and reasonable care by caregivers for ED patients.

**Plain English summary:**

The Family Coping Questionnaire has been used by researchers to assess the coping strategies of the relatives of patients. The present study aimed to validate a Japanese version of the Family Coping Questionnaire for Eating Disorders (FCQ-ED-J) and investigate the association between the coping strategies and psychological states of the caregivers of ED patients. Data from 150 caregivers, including 91 mothers and 34 fathers, were analyzed. The FCQ-ED-J comprised 13 items grouped into four subscales, with acceptable to good internal consistency (Cronbach’s alpha values between 0.71 and 0.85). All subscales of the FCQ-ED-J were found to be statistically significantly correlated with the profile of mood states (POMS) subscales. The Japanese caregivers’ responses to the patient’s ED symptom-related behavior were associated with their psychological states. Thus, the FCQ-ED-J can be utilized to help caregivers provide more effective and reasonable psychological care and support to ED patients.

## Background

Previous studies reported that family members of ED patients might suffer from emotional health problem as well as patients [1, 2]. Family members are often the main caregivers for ED patients. Caregivers has been defined as those who are the most concerned about the patient, supporting the patient's care at home, preparing the patient's meals, and attending hospital visits as significant persons [3]. Previous studies have reported that many caregivers for patients with ED symptoms suffer from depression and anxiety [3–16]. In addition, caregivers occasionally use maladaptive coping strategies, such as self‐blame, denial, and behavioral disengagement, to deal with their psychological burden, further amplifying their psychological distress [3, 17]. In Japan, the primary caregiver for ED patients is their mother, who spends the longest time with them [10, 18]. There are quite a few in-patient or day-patient treatment facilities in Japan that can provide specialized treatments for EDs [10, 19]. Therefore, caregivers living with the patient inevitably spend more time with the patient, a tendency that is even greater when the patient is in poor physical or mental condition [10].

The family coping questionnaire for eating disorders (FCQ-ED) was originally developed in Italy to assess the coping strategies of caregivers of patients with EDs [20]. Several previous studies of caregivers for ED patients showed that while task-oriented coping strategies were associated with lower levels of anxiety and depression [21, 22], emotion- and avoidance-oriented strategies were associated with higher distress [23, 24]. Although the family member is the primary caregiver and has more contact time with the ED patient, only a few studies have analyzed the relationship between ED patient caregivers’ coping strategies and their psychological states in the Japanese context [10, 18]. Furthermore, no equivalent questionnaire exists to evaluate such coping strategies.

In the present study, we developed a Japanese version of the FCQ-ED and evaluated its reliability and validity. A study of domestic caregivers of autism spectrum disorders reported higher psychological distress in Japanese caregivers than in Italian caregivers [25]. This may be related to the cultural background of Japan, which characterizes the collectivist society compared to Italy, which is more individualistic [25], and the social acceptability of emotionally negative expressions [26]. Therefore, the results of the present study could differ between the Japanese and Italian cultures. The present study aimed to validate the FCQ-ED for Japanese caregivers of patients with ED and examine the association between caregivers’ coping strategies and their psychological states. We hypothesize that FCQ-ED will extract features of Japanese ED patient caregivers’ coping strategies and the coping strategies will be associated with their psychological states.

## Methods

### Setting

The present study was approved by the Research Ethics Committee of the University of Tokyo. Recruitment was conducted from outpatients from December 2017 to December 2019, before the COVID-19 pandemic, at the Department of Stress and Psychosomatic Medicine in the University of Tokyo Hospital, in Tokyo, Japan. The present study was conducted in accordance with the Declaration of Helsinki and the “Ethical Guidelines for Medical and Health Research Involving Human Subjects” [10, 20, 27, 28].

### Participants

Because prolonged contact with a patient with ED is associated with poorer caregiver mental health, the participants had to have lived with and been actively involved in the care of a patient with ED [10]. The inclusion criteria for patients with EDs were as follows: (1) an ED diagnosis according to the Diagnostic and Statistical Manual of Mental Disorders, Fifth Edition (DSM-5) [29]; (2) aged between 16 and 65 years; and (3) lived with a caregiver for at least nine months during the last year, and continuously for the last three months. The inclusion criteria for caregivers were those aged between 20 and 65 years and actively involved in the patient’s care. Caregivers older than 65 years were excluded based on previous research because coping styles are influenced by age even when participant characteristics are used as controlled variables [20, 30, 31].

### Study procedure

First, we obtained permission to develop a Japanese version of the FCQ-ED methodology (FCQ-ED-J) from the corresponding author of the original version published in English [20]. The FCQ-ED was then translated into Japanese, and a final version was developed after further discussion and consensus among the authors, all of whom were native Japanese speakers and had expertise in the area of EDs. This version was back-translated into English by a professional bilingual editor, proficient in both English and Japanese and without expert knowledge of EDs, to confirm that the English and Japanese versions were conceptually equivalent. The first Japanese FCQ-ED translation required only minor changes.

Eligible ED patients and caregivers who visited the Department of Stress Sciences and Psychosomatic Medicine at The University of Tokyo Hospital received an explanation of the present study. The patients who agreed to have their caregivers participate in this study provided verbal informed consent. Additionally, the caregivers who willingly agreed to participate in this study provided written informed consent. More than one caregiver could be recruited per patient. The difference in the number of caregivers participating in the present study might have yielded a sampling bias. Participating caregivers were asked to complete the FCQ-ED-J and profile of mood states (POMS) [32] on paper at home or in the hospital waiting room. Following that, a chart review provided data on the patients’ and caregivers’ key sociodemographic and clinical characteristics.

### Measures

#### FCQ-ED

The FCQ-ED, which measures the coping strategies of caregivers of ED patients to the ED symptom-related behaviors, comprises 32 items grouped into six subscales: Coercion (i.e., angry and impulsive reactions to the patient’s behaviors), positive communication (i.e., calming and reassuring reactions), collusion with patient’s behaviors (i.e., not saying anything regarding the patient’s dysfunctional eating behaviors), seeking information (i.e., seeking advice on how to behave with the patient), avoidance (i.e., avoiding situations that are a reminder of the patient’s illness), and seeking spiritual help [20]. As in the original version, in this study, each item was rated on a four-point Likert scale (1: Never; 2: Rarely; 3: Sometimes; 4: Always). Higher subscale scores indicated more frequent application of the specific coping skill. The confirmatory factor analysis (CFA) in the original study identified a two-factor classification of coping strategies: Problem-oriented (coercion, collusion, avoidance) and emotion-focused (seeking information, positive communication) [20].

#### POMS

The caregivers’ psychological states were assessed using the POMS [32]. The POMS is a self-report instrument composed of six mood subscales (tension-anxiety, depression-dejection, anger-hostility, vigor, fatigue, and confusion) validated on clinical populations. The Japanese version has been regarded as adequately reliable and valid based on previous research indicating a Cronbach's alpha coefficient ranging between 0.779 and 0.926 for the mood subscales [33]. Vigor denotes a positive mood, while the others denote negative moods. Higher subscale scores for all but vigor indicate a poorer mood.

### Statistical analysis

The level of significance was set at 5% (*p* < 0.05). The analyses were performed using statistical package for the social sciences (SPSS) version 20.0 and SPSS Amos version 22.0.

### Reliability based on the original subscales

The reliability of each item for five of the FCQ-ED-J subscales was measured using Cronbach’s alpha values to assess the internal consistency of the instrument [34, 35]. The spiritual help subscale (one of the six subscales of the original FCQ-ED) was excluded because it comprised only one item.

### Confirmatory factor analysis

To confirm the factor structure of the FCQ-ED-J, we ran a CFA to test the fit of the higher-order factor model that was reported [9]. The extracted factors were used as latent variables in the CFA, while the items belonging to them were used as observation variables, followed by a covariance structure analysis. The comparative fit index (CFI) and the root mean square error of approximation (RMSEA) were used to evaluate model adequacy [36, 37].

### Exploratory factor analysis

To reconsider the underlying factor structure in the FCQ-ED-J, exploratory factor analysis (EFA) was performed as the original five-subscale model did not indicate a good fit. The Kaiser–Meyer–Olkin (KMO) and Bartlett’s test of sphericity values verified the appropriateness of the factor analysis. The number of factors was determined by the scree plot of the factors and the cumulative contribution rate before rotation. The EFA was performed by the maximum likelihood and promax rotation methods, with a factor loading of 0.50 or more as a reference [38], based on the Kaiser–Guttman criterion that meaningful factors should be associated with eigenvalues greater than 1.0 [39, 40].

### Reliability based on the new subscales

The reliability of each item on the new FCQ-ED-J subscales was also measured with Cronbach’s alpha to assess the internal consistency of the instrument [34, 35].

### Validity

Concurrent validity for the FCQ-ED-J and POMS was assessed using Pearson’s correlation coefficients. The evaluations of the Pearson’s correlation coefficients were based on Cohen’s criteria (0.10 ≤ *r* < 0.3: weak correlation, 0.3 ≤ *r* < 0.5: moderate correlation, *r* ≥ 0.5: strong correlation) [41]. Multivariate analysis after adjusting for potential confounding factors was not performed in the present study.

## Results

### Participants

One hundred seventy eligible caregivers received the explanation of this study. Of these, 154 caregivers completed FCQ-ED-J (90.6%), of which eight caregivers refused to participate; five caregivers’ patients were transferred to other hospitals or stopped visiting; two caregivers lost the FCQ-ED-J, and one caregiver did not submit the FCQ-ED-J. Four caregivers had to be excluded owing to missing data, leaving 150 caregivers to be included in the study.

We obtained data from 109 patients (107 female, 2 male). The patients’ diagnoses were as follows: 56 diagnosed with anorexia nervosa restricting type (AN-R), 40 with anorexia nervosa binge‐eating/purging (AN-BP), 10 with bulimia nervosa (BN), 2 with binge-eating disorder (BED), and 1 with other ED.

The participants (98 female, 52 male) consisted of 150 caregivers (91 mothers, 34 fathers, 14 spouses, 7 siblings, 3 children, and 1 partner); the distribution was as follows: 80 AN-R (53.3%), 52 AN-BP (35.7%), 15 BN (10.0%), 2 BED (1.3%), and 1 other ED (0.7%). The mean age of the caregivers was 51.1 ± 12.0 years. The sociodemographic characteristics of the patients and caregivers are reported in Table 1.

**Table 1:** Socio-demographic and clinical characteristics

Patients' socio-demographic and clinical characteristics (*N* = 109)	
Gender, F, % (*N*)	98.2 (107)
Age, *M* (*SD*)	24.6 (9.6)
Duration of the illness, months, *M* (*SD*)	70.8 (77.9)
BMI, kg/m^2^, *M* (*SD*)	15.6 (3.9)
Age of onset, *M* (*SD*)	18.6 (7.2)
Caregivers' socio-demographic characteristics (*N* = 150)	
Gender, F, % (*N*)	65.3 (98)
Age, *M* (*SD*)	51.1 (12.0)
Relationship with the patient, % (*N*)	
Mother	60.7 (91)
Father	22.7 (34)
Spouse	9.3 (14)
Sibling	4.7 (7)
Child	2.0 (3)
Partner	0.7 (1)
Diagnosis of patients being cared for by Caregivers (*N* = 150), % (*N*)	
Anorexia nervosa restricting type (AN-R)	53.3 (80)
Anorexia nervosa binge-eating/purging (AN-BP)	34.7 (52)
Bulimia nervosa (BN)	10.0 (15)
Binge-eating disorder (BED)	1.3 (2)
Others	0.7 (1)

### Reliability of the original FCQ-ED subscales

The Cronbach’s alpha values ranged between 0.66 and 0.45 for the original five FCQ-ED subscales (coercion, 0.66; positive communication, 0.62; collusion, 0.45; seeking information, 0.50; avoidance, 0.46).

### Confirmatory factor analysis

CFA results for the original five-subscale model indicated inadequate model fit (CFI = 0.41; RMSEA = 0.10) [36, 37]. Therefore, the underlying factor structure of the FCQ-ED-J was reconsidered using EFA.

### Exploratory factor analysis

**Fig. 1:**
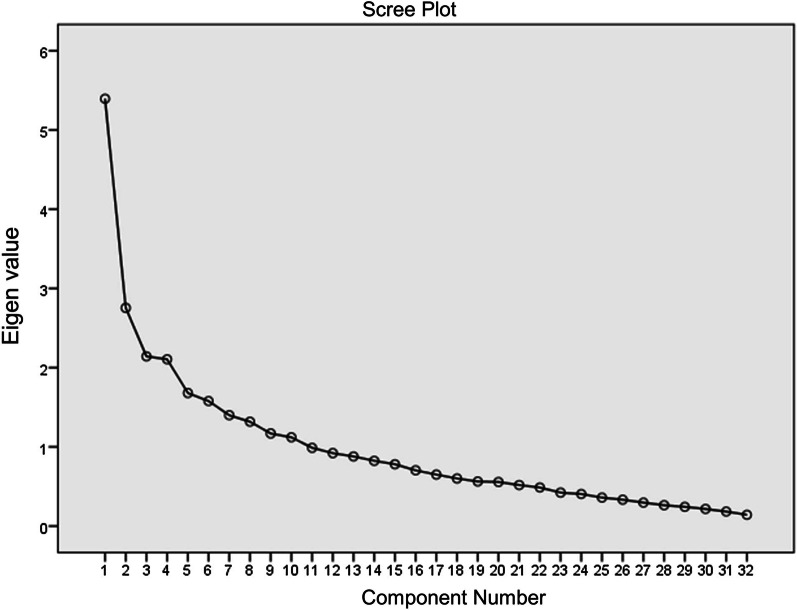
Scree plot of conducting an exploratory factor analysis on the FCQ-ED-J. Scree plot depicting eigen values of 32 components of FCQ-ED-J. FCQ-ED-J, Family Coping Questionnaire for Eating Disorders

The KMO test confirmed moderate sampling adequacy (0.68). In addition, Bartlett’s test of sphericity yielded statistically significant results (*p* < 0.01), indicating that the correlation matrix was suitable for factor analysis. In this model, four factors, representing 30.89% of the total variance, were extracted based on eigenvalues ≥ 1 and the scree plot (Fig. 1) [39, 40]. The 13 items in the four-factor solution were considered appropriate by examining the magnitude and rate of change in eigenvalues. Promax rotation was used for the factor solution interpretation (Table 2). Items with factor loadings < 0.5 and those with more than one were excluded [38].

**Table 2:** Factor loadings of FCQ-ED-J following exploratory factor analysis with maximum-likelihood estimation and promax rotation; and subscales of the FCQ-ED-J and the reliability

FCQ-ED item	Factor 1	Factor 2	Factor 3	Factor 4
	Response to binge-eating	Response to frequent weighing	Response to too much physical exercise	Response to abusing laxatives and/or diuretics
When I saw that S was trying to avoid eating a lot, I told him/her that I was glad and encouraged him/her to do so. (PC)	**0.73**	− 0.04	− 0.04	− 0.12
I managed to keep calm even when S had emptied the fridge or the cupboard or bought too much food on his/her own. (PC)	**0.63**	− 0.08	0.01	0.13
I hid the food to S to prevent he/she ate too much. (CE)	**0.51**	0.06	− 0.01	− 0.07
When I saw that S was preparing large amounts of food to eat, I got angry and told him/her off. (CE)	**0.50**	− 0.03	0.27	− 0.06
When S checked his/her body weight too frequently, I shouted. (CE)	− 0.05	**0.84**	0.03	− 0.05
When S has frequently checked his/her weight, I calmly tried to convince him/her not to do it again. (PC)	− 0.06	**0.76**	− 0.02	0.05
When S checked his/her body weight too frequently, I hid the weight scale. (CE)	− 0.002	**0.70**	0.04	− 0.02
When S has frequently checked his/her weight, I did not say anything and pretended nothing had happened. (CL)	0.07	**0.70**	− 0.06	0.10
When S checked his/her body weight too frequently, I reacted angrily. (CE)	0.03	**0.61**	0.06	0.11
When S has done too much physical exercise, I tried calmly to convince him/her not to do it again. (PC)	− 0.12	0.05	**0.90**	− 0.04
When S has done too much physical exercise, I did not say anything and pretended nothing had happened. (CL)	− 0.14	− 0.08	**0.85**	0.06
When S has abused laxatives and/or diuretics, I did not say anything and pretended nothing had happened. (CL)	0.01	0.006	− 0.02	**0.90**
When S has abused laxatives and/or diuretics, I calmly tried to convince him/her not to do it again. (PC)	0.08	0.07	0.10	**0.66**
When I saw that S was vomiting, I reacted angrily. (CE)	0.46	− 0.002	0.004	0.15
When I saw that S was eating everything that came to hand very quickly, I reacted angrily. (CE)	0.44	0.07	0.06	− 0.04
When S did not want to meet the doctor, I preferred not to force him/her. (CL)	0.41	0.01	− 0.11	0.03
When I saw that S was vomiting, I calmly tried to convince him/her not to do it again. (PC)	0.40	0.05	− 0.07	0.22
When I saw that S got locked in the bathroom to vomit, I shouted him/her to get out. (CE)	0.38	− 0.02	− 0.09	0.05
When S has refused to take medication and/or to meet the psychiatrist or psychologist, I have not done anything to make him/her change his/her. (CL)	0.37	0.04	− 0.03	− 0.08
When I saw that S was alone, I tried to get him/her to meet his/her friends. (PC)	0.34	0.28	0.05	− 0.30
Whenever S was nervous or anxious, I asked him to sit with me and tell me what was wrong and I tried to reassure him/her. (PC)	0.29	− 0.18	0.19	− 0.07
When S was aggressive with me, I managed to keep calm. (PC)	0.19	0.08	0.14	− 0.06
I prayed and I confided to a priest so that the situation would improve. (SH)	0.16	0.11	− 0.02	− 0.01
I tried to get information on S’ disorder. (SI)	− 0.05	− 0.24	− 0.23	− 0.14
I managed to stay calm even when S had eaten little or nothing all day. (PC)	0.04	0.10	0.38	− 0.02
I sought advice on how to behave with S. (SI)	− 0.06	− 0.18	− 0.22	0.04
I avoided to have lunch alone with S. (A)	− 0.16	− 0.03	− 0.17	− 0.02
I met with relatives and friends to avoid thinking about S situation. (A)	− 0.10	− 0.04	− 0.16	0.15
I looked for new interests to avoid thinking about S situation. (A)	− 0.13	0.04	− 0.15	0.08
When I saw that S was vomiting, I did not say anything and pretended nothing had happened. (CL)	0.34	− 0.07	− 0.03	0.35
After every meal, I lock the bathroom to prevent S from vomiting. (CE)	− 0.12	0.07	− 0.12	0.34
I avoided to have lunch with friends or relatives in the presence of S. (A)	− 0.05	− 0.07	− 0.01	− 0.11
Cronbach's alpha	**0.71**	**0.85**	**0.85**	**0.79**

Exploratory analysis with promax rotation was used to interprete the factor solusion. This four-factor solution was considered appropriate by examining the magnitude and rate of change in Eigen values. Original subscale allocations are shown in brackets. Bold values in this table show that they are included in "Factor 1", "Factor 2", "Factor 3", and "Factor 4" columns respectively

*CE* coercion, *PC* positive communication, *CL* collusion, *SI* seeking information, *A* avoidance, *SH* spiritual help

Using EFA, we obtained four important factors: “response to binge-eating” (factor 1), “response to frequent weighing” (factor 2), “response to too much physical exercise” (factor 3), and “response to abusing laxatives and/or diuretics” (factor 4). Contrary to the original FCQ-ED subscales, which corresponded to caregivers’ coping strategies, the FCQ-ED-J subscales were interpreted as patients’ behavioral categories based on the items.

### Reliability based on the four new subscales

The Cronbach’s alpha coefficients for the four new subscales—0.71 for “response to binge-eating” (factor 1), 0.85 for “response to frequent weighing” (factor 2), 0.85 for “response to too much physical exercise” (factor 3), and 0.79 for “response to abusing laxatives and/or diuretics” (factor 4) (Table 2)—were indicative of acceptable to good internal consistency [42, 43].

### Validity

The correlations between each FCQ-ED-J subscale and the six POMS mood subscales (Table 3) were as follows. Response to binge-eating was statistically significantly positively correlated with anger-hostility (*r* = 0.21, *p* = 0.013); response to frequent weighing was statistically significantly positively correlated with tension-anxiety (*r* = 0.19, *p* = 0.024) and anger-hostility (*r* = 0.19, *p* = 0.023); response to too much physical exercise was positively correlated with almost all subscales (*r* = 0.20–0.32, *p* = 0.000–0.016), except vigor (*r* =  − 0.14, *p* = 0.095); and response to abusing laxatives and/or diuretics was statistically significantly positively correlated with tension-anxiety (*r* = 0.17, *p* = 0.039) and depression-dejection, (*r* = 0.27, *p* = 0.001) and negatively correlated with vigor (*r* =  − 0.21, *p* = 0.012).

**Table 3:** The Correlations between the FCQ-ED-J and the POMS (*p* < 0.05)

POMS	T-A	D	A-H	V	F	C
*FCQ-ED-J*						
Response to binge-eating	0.13	0.16	0.21*	0.02	0.11	0.12
Response to frequent weighing	0.19*	0.09	0.19*	0.01	0.08	0.12
Response to too much physical exercise	0.24**	0.20*	0.20*	− 0.14	0.27**	0.32***
Response to abusing laxatives and/or diuretics	0.17*	0.27**	0.13	− 0.21*	0.07	0.12

*T–A* tension–anxiety, *D* depression–dejection, *A–H* anger–hostility, *V* vigor, *F* fatigue, *C* confusion

* < 0.05, ** < 0.01, *** < 0.001

With regard to the required sample sizes (power analysis), 85 and 194 participants were estimated to be enough to detect correlation coefficients of 0.3 and 0.2 respectively at 5% level of the type I error with 80% power [41].

## Discussion

This study aimed to validate the FCQ-ED for Japanese caregivers of ED patients and examine the association between caregivers’ coping strategies and their psychological states. We developed a Japanese version of the FCQ-ED (FCQ-ED-J) and confirmed its reliability and validity.

The CFA in the present study did not support the original structure. The EFA revealed that the FCQ-ED-J comprised a 13-item scale with four factors: “response to binge-eating” (factor 1), “response to frequent weighing” (factor 2), “response to too much physical exercise” (factor 3), and “response to abusing laxatives and/or diuretics” (factor 4), all with acceptable to good ranges of calculated Cronbach’s alphas, despite the limited number of items in each category. Binge-eating, frequent weighing, too much physical exercise, and the abuse of laxatives and/or diuretics are almost uncontrollable behaviors for caregivers. Japanese caregivers for ED patients may have similar responses to these uncontrollable behaviors. Dysregulated ED behaviors have been positively and significantly associated with the burden of care for ED patients [44]. Previous studies have shown that increasing the burden of long-term care exacerbated the psychological burden on the caregivers of ED patients [10, 15]. Therefore, the present study’s factor structure of the FCQ-ED-J can explain the relationship between dysregulated ED behaviors, difficulty in providing care, and the psychological burden of caregivers. Also, intrusive symptoms of an ED can trigger emotionally-driven responses such as criticism, hostility, and over-protectiveness in caregivers [45].

Three subscales of the FCQ-ED-J (“response to frequent weighing,” “response to too much physical exercise,” and “response to abusing laxatives and/or diuretics”) were weakly positively correlated with tension-anxiety [46, 47]. Additionally, two FCQ-ED-J subscales (“response to too much physical exercise” and “response to abusing laxatives and/or diuretics”) were weakly positively correlated with depression-dejection. In previous findings, parents of patients with AN presented with higher levels of anxiety and depression [21]. Although we did not compare our data with that of the healthy control group, our results were consistent with the finding that caregivers for ED patients were more likely to report anxiety and depression [3–16]. Our findings regarding the relationships between “response to abusing laxatives and/or diuretics” and psychological distress substantiate several previous studies, which found that caregivers of patients who purge reported a stronger burden of care [1, 48]. Specifically, “response to too much physical exercise” was highly correlated with almost all POMS subscales in the present study. Previous research has shown that nutrition is the best predictor of anxiety and depression [2]. Considering the large number of AN caregivers included in our sample (88%), excessive exercise by patients with low body weight or inadequate nutrition may increase the psychological burden of their caregivers.

Several studies have suggested that the emotional reactions of caregivers to EDs (e.g., high levels of stress, anxiety, depression, and hostility) resulted in perpetuating EDs [49, 50]. In our study, “response to binge-eating,” “response to frequent weighing,” and “response to too much physical exercise” were also positively correlated with anger-hostility. According to a previous ED caregivers’ study, mothers who felt that they received higher levels of social support felt less isolated and/or depressed [18], and affective support was significantly associated with decreased severity of the patients’ symptoms [10]. The FCQ-ED-J can act as an effective tool for detecting the psychological burden of caregivers and increasing social support for them. Healthcare providers can discuss the 13 coping strategies identified in the FCQ-ED-J, and share the associations between these coping strategies and their psychological burden with caregivers.

The present study is one of the few to investigate the psychological characteristics of caregivers of patients with EDs in Japan. This study is also the first to develop the Japanese version of the FCQ-ED and demonstrate the relationship between Japanese caregivers’ responses to ED behaviors and their psychological states.

This study was also subject to some limitations. First, the number of male participants in the study sample was low, and our sample size was not enough to detect a weak correlation coefficient. A larger sample size is also necessary to extract gender- or relation-specific differences. Second, the FCQ-ED-J was evaluated using a self-reported instrument, and the participants’ responses may have been influenced by the social desirability effect or an underreporting bias [51, 52]. For example, during the present study, a patient reported that her parents usually accused her of binge-eating and abusing laxatives; however, her parents did not reveal the same in their FCQ-ED-J responses. Third, caregivers’ demographics including, education level, occupation, or income, were not collected. We have not performed multivariate analysis after adjusting for potential confounding factors in the present study.

In order to improve these limitations, we aim to perform a large sample size study involving more male caregivers. It is necessary to obtain actual ED behaviors, and the demographic data from not only patients but also caregivers. Future study is needed to investigate how ED behaviors and caregiving directly affects the caregiver's mental health using a scale that measures the burden of caregiving.

## Conclusions

To summarize, the FCQ-ED-J utilized four new factors, different from the original FCQ-ED methodology, and demonstrated sufficient reliability and validity. The FCQ-ED-J can help healthcare providers and caregivers understand what caregivers’ responses to ED behaviors make their psychological burden. Therefore, the FCQ-ED-J may be used as one of the means of providing more effective and reasonable care by the caregivers for patients with ED by providing the chance to consider other coping strategies to ED behaviors.

## Data Availability

The datasets generated and/or analyzed during the current study are not publicly available due to ethical restrictions, but are available from the corresponding author on reasonable request.

## Abbreviations

FCQ-ED: Family coping questionnaire for eating disorders
FCQ-ED-J: Japanese version of the family coping questionnaire for eating disorders
POMS: Profile of mood states
ED: Eating disorder
DSM-5: Diagnostic and statistical manual of mental disorders, fifth edition
CFA: Confirmatory factor analysis
CFI: Comparative fit index
RMSEA: Root mean square error of approximation
EFA: Exploratory factor analysis
KMO: Kaiser–Meyer–Olkin
AN-R: Anorexia nervosa restricting type
AN-BP: Anorexia nervosa binge-eating/purging
BN: Bulimia nervosa
BED: Binge-eating disorder

## References

[1] Martín J, Padierna A, Aguirre U, Quintana JM, Hayas CL, Muñoz P (2011). Quality of life among caregivers of patients with eating disorders. Qual Life Res.

[2] Sepúlveda AR, Graell M, Berbel E, Anastasiadou D, Botella J, Carrobles JA, Morandé G (2012). Factors associated with emotional well-being in primary and secondary caregivers of patients with eating disorders. Eur Eat Disord Rev.

[3] Treasure J, Nazar BP (2016). Interventions for the carers of patients with eating disorders. Curr Psychiatry Rep.

[4] Baronet AM (1999). Factors associated with caregiver burden in mental illness: a critical review of the research literature. Clin Psychol Rev.

[5] Coomber K, King RM (2012). Coping strategies and social support as predictors and mediators of eating disorder carer burden and psychological distress. Soc Psychiatry Psychiatr Epidemiol.

[6] Coomber K, King RM (2013). A longitudinal examination of burden and psychological distress in carers of people with an eating disorder. Soc Psychiatry Psychiatr Epidemiol.

[7] Fleischmann H, Klupp A (2004). Zur Lebensqualität der Angehörigen psychisch Kranker [Quality of life in relatives of mentally ill people]. Psychiatr Prax.

[8] Guethmundsson OO, Tómasson K (2002). Quality of life and mental health of parents of children with mental health problems. Nord J Psychiatry.

[9] Kyriacou O, Treasure J, Schmidt U (2008). Expressed emotion in eating disorders assessed via self-report: an examination of factors associated with expressed emotion in carers of people with anorexia nervosa in comparison to control families. Int J Eat Disord.

[10] Ohara C, Komaki G, Yamagata Z, Hotta M, Kamo T, Ando T (2016). Factors associated with caregiving burden and mental health conditions in caregivers of patients with anorexia nervosa in Japan. Biopsychosoc Med.

[11] Santonastaso P, Saccon D, Favaro A (1997). Burden and psychiatric symptoms on key relatives of patients with eating disorders: a preliminary study. Eat Weight Disord.

[12] Highet N, Thompson M, King RM (2005). The experience of living with a person with an eating disorder: the impact on the carers. Eat Disord.

[13] Treasure J, Murphy T, Szmukler G, Todd G, Gavan K, Joyce J (2001). The experience of caregiving for severe mental illness: a comparison between anorexia nervosa and psychosis. Soc Psychiatry Psychiatr Epidemiol.

[14] Whitney J, Haigh R, Weinman J, Treasure J (2007). Caring for people with eating disorders: factors associated with psychological distress and negative caregiving appraisals in carers of people with eating disorders. Br J Clin Psychol.

[15] Zabala MJ, Macdonald P, Treasure J (2009). Appraisal of caregiving burden, expressed emotion and psychological distress in families of people with eating disorders: a systematic review. Eur Eat Disord Rev.

[16] Martín J, Padierna A, Aguirre U, González N, Muñoz P, Quintana JM (2013). Predictors of quality of life and caregiver burden among maternal and paternal caregivers of patients with eating disorders. Psychiatry Res.

[17] Goddard E, Salerno L, Hibbs R, Raenker S, Naumann U, Arcelus J, Ayton A, Boughton N, Connan F, Goss K, Lacey H, Laszlo B, Morgan J, Moore K, Robertson D, Schreiber-Kounine C, Sharma S, Whitehead L, Schmidt U, Treasure J (2013). Empirical examination of the interpersonal maintenance model of anorexia nervosa. Int J Eat Disord.

[18] Yamada A, Katsuki F, Kondo M, Sawada H, Watanabe N, Akechi T (2021). Association between the social support for mothers of patients with eating disorders, maternal mental health, and patient symptomatic severity: a cross-sectional study. J Eat Disord.

[19] Hotta M, Horikawa R, Mabe H, Yokoyama S, Sugiyama E, Yonekawa T (2015). Epidemiology of anorexia nervosa in Japanese adolescents. Biopsychosoc Med.

[20] Fiorillo A, Sampogna G, Del Vecchio V, Luciano M, Monteleone AM, Di Maso V, Garcia CS, Barbuto E, Monteleone P, Maj M (2015). Development and validation of the family coping questionnaire for eating disorders. Int J Eat Disord.

[21] Kyriacou O, Treasure J, Schmidt U (2008). Understanding how parents cope with living with someone with anorexia nervosa: modelling the factors that are associated with carer distress. Int J Eat Disord.

[22] Troop NA, Holbrey A, Trowler R, Treasure JL (1994). Ways of coping in women with eating disorders. J Nerv Ment Dis.

[23] Shatford LA, Evans DR (1986). Bulimia as a manifestation of the stress process: LISREL causal modeling analysis. Int J Eat Disord.

[24] Endler NS, Parker JDA, Butcher JN (1993). A factor analytic study of coping styles and the MMPI-2 content scales. J Clin Psychol.

[25] Esposito G, Nakazawa J, Venuti P, Bornstein MH (2012). Perceptions of distress in young children with autism compared to typically developing children: a cultural comparison between Japan and Italy. Res Dev Disabil.

[26] Hughes C, Devine R, Ensor R, Koyasu M, Mizokawa A, Lecce S (2014). Lost in translation? Comparing British, Japanese, and Italian Children’s theory-of-mind performance. Child Dev Res.

[27] World Medical Association (2013). World Medical Association Declaration of Helsinki: ethical principles for medical research involving human subjects. JAMA.

[28] Sone S (2015). Ethical guidelines for clinical trials in medical research involving human subjects. Gan To Kagaku Ryoho.

[29] American Psychiatric Association. Diagnostic and statistical manual of mental disorders: DSM-5. Arlington: American Psychiatric Association; 2013.

[30] Felton BJ, Revenson TA (1987). Age differences in coping with chronic illness. Psychol Aging.

[31] Fiorillo A, Sampogna G, Luciano M (2017). How do relatives cope with eating disorders? Results from an Italian multicentre study. Int J Eat Disord.

[32] McNair DM, Loor M, Droppleman LF. Profile of mood states. San Diego: Educational and Industrial Testing Service; 1981.

[33] Yokoyama K, Araki S, Kawakami N, Tkakeshita T. [Production of the Japanese edition of profile of mood states (POMS): assessment of reliability and validity]. *Nihon Koshu Eisei Zasshi*.1990;37(11):913–8. 2132363

[34] Cortina JM (1993). What is coefficient alpha? An examination of theory and applications. J Appl Soc Psychol.

[35] Bland JM, Altman DG (1997). Cronbach's alpha. BMJ.

[36] Basto M, Pereira JM (2012). An SPSS R-menu for ordinal factor analysis. J Stat Softw.

[37] Hu L, Bentler MP (1999). Cutoff criteria for fit indexes in covariance structure analysis: conventional criteria versus new alternatives. Struct Equ Model.

[38] Engel SG, Wittrock DA, Crosby RD, Wonderlich SA, Mitchell JE, Kolotkin RL (2006). Development and psychometric validation of an eating disorder-specific health-related quality of life instrument. Int J Eat Disord.

[39] Gorgush RL (1978). Psychometric theory.

[40] Harman HH (1967). Modern factor analysis.

[41] Cohen J (1988). Statistical power analysis for the behavioral sciences.

[42] Cronbach LJ (1951). Coefficient alpha and the internal structure of tests. Psychomerika.

[43] Tavakol M, Dennick R (2011). Making sense of Cronbach's alpha. Int J Med Educ.

[44] Sepulveda AR, Whitney J, Hankins M, Treasure J (2008). Development and validation of an Eating Disorders Symptom Impact Scale (EDSIS) for carers of people with eating disorders. Health Qual Life Outcomes.

[45] Anastasiadou D, Medina-Pradas C, Sepulveda AR, Treasure J (2014). A systematic review of family caregiving in eating disorders. Eat Behav.

[46] Akoglu H (2018). User's guide to correlation coefficients. Turk J Emerg Med.

[47] Schober P, Boer C, Schwarte LA (2018). Correlation coefficients: appropriate use and interpretation. Anesth Analg.

[48] Sepulveda AR, Anastasiadou D, Pellegrin Y, Andrés P, Graell M, Carrobles JA, Morandé G (2014). Impact of caregiving experience on mental health among caregivers: a comparison of eating disorder patients with purging and non-purging behaviors. Eat Weight Disord.

[49] Treasure J, Rhind C, Macdonald P, Todd G (2015). Collaborative care: the new Maudsley model. Eat Disord.

[50] Treasure J, Willmott D, Ambwani S, Cardi V, Clark Bryan D, Rowlands K, Schmidt U (2020). Cognitive interpersonal model for anorexia nervosa revisited: the perpetuating factors that contribute to the development of the severe and enduring illness. J Clin Med.

[51] Bou Malham P, Saucier G (2016). The conceptual link between social desirability and cultural normativity. Int J Psychol.

[52] Lanyon RI, Wershba RE (2013). The effect of underreporting response bias on the assessment of psychopathology. Psychol Assess.

